# A curated dataset of complete Enterobacteriaceae plasmids compiled from the NCBI nucleotide database

**DOI:** 10.1016/j.dib.2017.04.024

**Published:** 2017-04-23

**Authors:** Alex Orlek, Hang Phan, Anna E. Sheppard, Michel Doumith, Matthew Ellington, Tim Peto, Derrick Crook, A. Sarah Walker, Neil Woodford, Muna F. Anjum, Nicole Stoesser

**Affiliations:** a Nuffield Department of Medicine, University of Oxford, John Radcliffe Hospital, Oxford, UK; b NIHR Health Protection Research Unit in Healthcare Associated Infections and Antimicrobial Resistance, University of Oxford, Oxford, UK; c Antimicrobial Resistance and Healthcare Associated Infections (AMRHAI) Reference Unit, National Infection Service, Public Health England, London, UK; d Department of Bacteriology, Animal and Plant Health Agency, Addlestone, UK

**Keywords:** Plasmids, Sequence data curation, Complete genomes, Enterobacteriaceae family

## Abstract

Thousands of plasmid sequences are now publicly available in the NCBI nucleotide database, but they are not reliably annotated to distinguish complete plasmids from plasmid fragments, such as gene or contig sequences; therefore, retrieving complete plasmids for downstream analyses is challenging. Here we present a curated dataset of complete bacterial plasmids from the clinically relevant Enterobacteriaceae family. The dataset was compiled from the NCBI nucleotide database using curation steps designed to exclude incomplete plasmid sequences, and chromosomal sequences misannotated as plasmids. Over 2000 complete plasmid sequences are included in the curated plasmid dataset. Protein sequences produced from translating each complete plasmid nucleotide sequence in all 6 frames are also provided. Further analysis and discussion of the dataset is presented in an accompanying research article: “Ordering the mob: insights into replicon and MOB typing…” (Orlek et al., 2017) [1]. The curated plasmid sequences are publicly available in the Figshare repository.

**Specifications Table**

**Table:** 

Subject area	Microbiology, Bioinformatics
More specific subject area	Plasmids
Type of data	Sequence data
How data was acquired	Plasmid nucleotide sequences were compiled from Genbank and RefSeq accessions contained within the NCBI nucleotide database. Corresponding protein sequences were generated by translating each plasmid nucleotide sequence in all 6 frames.
Data format	FASTA files, Genbank files (zipped)
Experimental factors	N/A
Experimental features	N/A
Data source location	Sequences were retrieved from the NCBI nucleotide database (https://www.ncbi.nlm.nih.gov/nucleotide/); geographic location metadata was not retrieved.
Data accessibility	Data is publicly available in the Figshare repository.
	https://figshare.com/s/18de8bdcbba47dbaba41
	DOI: D10.6084/m9.figshare.4609303

**Value of the data**

• To our knowledge, this is currently the only large curated dataset of complete plasmids, compiled according to well-defined, and transparently validated, inclusion and exclusion criteria.
• The data could be used to benchmark the performance of plasmid typing schemes [1].
• The data could be used for reference-based plasmid analyses [2]; for example, contigs could be queried against the curated plasmid sequences with the aim of distinguishing plasmid from chromosomal contigs [3] or assessing plasmid genetic content [4].
• The protein dataset is a useful resource for MOB typing [5]. Information about sequence conservation from aligned protein database sequences can be harnessed using more powerful profile-based homology searching [6], enabling improved MOB typing compared with standard protein BLAST. A bioinformatic protocol and code for MOB typing using the protein dataset are provided on GitHub (https://github.com/AlexOrlek/MOBtyping).
• Those interested in the epidemiology of plasmid-mediated antibiotic resistance in the Enterobacteriaceae family could use the data to extend previous analyses [1].

## 1 Data

The data consists of nucleotide sequences of 2097 complete Enterobacteriaceae plasmids, compiled from the NCBI nucleotide database (‘nucleotideseq.fa’). In addition, we provide a corresponding dataset of 12,582 protein sequences (‘translatedproteinseq.fa’), derived from translating each plasmid nucleotide sequence in all 6 frames. Nucleotide and protein sequence datasets are formatted as FASTA files. Headers in the protein FASTA file are in the following format: >accession id|strand|frame|protein sequence length. Furthermore, NCBI Genbank files, with detailed information on accessions, are also provided. One Genbank file contains the 2097 complete curated plasmid accessions (‘filtered_2097plasmids.gb.gz’). Another Genbank file contains 6952 accessions (‘6952plasmids.gb.gz’), obtained using an initial query, prior to removing duplicate sequences or applying inclusion/exclusion criteria.

## 2 Experimental design, materials and methods

Putative complete plasmid accessions were retrieved from the NCBI nucleotide database (https://www.ncbi.nlm.nih.gov/nucleotide/) on 26th August 2016, using an Entrez query with filters to exclude some incomplete or non-plasmid accessions at this stage. Following this initial query, duplicate sequences (those sharing 100% nucleotide sequence identity with another retrieved sequence) were removed. Biopython scripts [7] were used to filter-out non-coding sequences. Regular expression searches of accession title descriptions were used to apply exclusion and inclusion criteria. Subsequent filtering involved conducting multi-locus sequence typing (MLST) to exclude chromosomal accessions misannotated as plasmids. In addition, the ‘completeness’ annotation (included as accession metadata in NCBI) was used to further exclude partial plasmid sequences. Additional filtering involved manual inspection of putative plasmids at the tails of the sequence length distribution, to remove remaining accessions that represented chromosomal sequences or partial plasmid sequences. A more detailed description of these methods can be found in the accompanying research article [1].
